# Evaluation of *Pediococcus acidilactici* AS185 as an adjunct culture in probiotic cheddar cheese manufacture

**DOI:** 10.1002/fsn3.3198

**Published:** 2022-12-23

**Authors:** Chao Wang, Lei Gao, Yansong Gao, Ge Yang, Zijian Zhao, Yujuan Zhao, Jihui Wang, Shengyu Li

**Affiliations:** ^1^ School of Biological Engineering Dalian Polytechnic University Dalian China; ^2^ Institute of Agro‐food Technology Jilin Academy of Agricultural Sciences/National R&D Center for Milk Processing Changchun China; ^3^ Engineering Research Center of Health Food Design & Nutrition Regulation, School of Chemical Engineering and Energy Technology Dongguan University of Technology Dongguan China

**Keywords:** cheddar cheese, FAAs, *Pediococcus acidilactici*, physicochemical properties, probiotics, SCFAs, sensory acceptance, textural properties

## Abstract

A novel probiotic *Pediococcus acidilactici* AS185, isolated from traditional Chinese fermented foods, was used as an adjunct culture for probiotic cheddar cheese production. The physicochemical composition, textural, free amino acids (FAAs), short‐chain fatty acids (SCFAs) profiles, sensory properties, and microbial survival, was evaluated during the 90‐day ripening period. The addition of *P. acidilactici* AS185 did not influence the physicochemical composition of cheddar cheese but significantly decreased the hardness without affecting its textural profile. During ripening, *P. acidilactici* AS185 was able to grow and promote the generation of FAAs and SCFAs, but did not alter the overall sensory properties; it rather improved the flavor and taste of cheese. In addition, the cheese matrix protected strain *P. acidilactici* AS185 during transit throughout the simulated gastrointestinal system. These results demonstrated that *P. acidilactici* AS185 adjunct cultures might be useful for producing high‐quality probiotic cheddar cheese.

## Graphical abstract

*Pediococcus acidilactici* AS185 promote the generation of FAAs and SCFAs during ripening of cheddar cheese. *P. acidilactici* AS185 did not alter the overall sensory properties rather improved the flavor and taste of cheese.

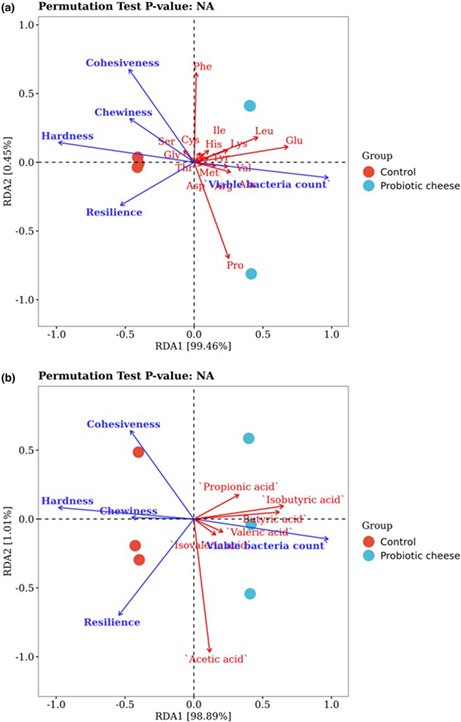

## 1 INTRODUCTION

Probiotic foods offer health benefits based on live probiotic bacterial content that maintains probiotic viability throughout food production (initial inoculation, processing, and storage steps) and during the passage of the ingested organisms through the gastrointestinal tract (Sagheddu et al., 2018; Villena & Kitazawa, 2017). After consumption of probiotic foods, the probiotic organisms must attach to the host to ensure their persistence in the body for long‐term beneficial effects. Typically, yogurt and other fermented milks are the most common foods used to deliver probiotic bacteria, though some studies have revealed that the characteristics of these foods may compromise the viability of probiotic organisms (Ng et al., 2010; Sanders & Marco, 2010; Vinderola et al., 2000). Conversely, cheese (such as gouda, white, and cheddar cheese) have several advantages over fermented milk as a deliver system for viable probiotic microorganisms, such as high‐fat content, high pH value, strong buffering ability, low titratable acidity and oxygen content, dense texture, and other physical and chemical properties (Hammam & Ahmed, 2019). Several studies have highlighted fresh and cheddar cheese varieties as excellent carriers of probiotic bacteria (Banks & Williams, 2004; Buriti et al., 2005; Karimi et al., 2011; Ong et al., 2006, 2007; Vinderola et al., 2000). Furthermore, as probiotic carriers, cheese varieties shield the probiotic organisms from exposure to harsh environmental conditions during production, ripening, and storage, as well as during passage through the gastrointestinal tract allowing a high number of viable bacteria to arrive at the target sites within the body to exert beneficial health effects (Stanton et al., 1998).

Probiotic cheese varieties, defined as ripened or unripened cheese products that provide human health benefits, are created by the addition of probiotics as auxiliary fermentation agents to pasteurized raw milk or curd after pressing, whey removal, and addition of main fermentation agents (da Cruza et al., 2009). These added probiotic adjunct cultures influence the texture and flavor of cheese production and ripening. These effects originate from probiotic proteases, lipases, or other microbial enzymes or metabolites (e.g., lactases, extracellular polysaccharides, antibacterial compounds, and downstream functional components), which collectively influence the flavor and quality, as well as the structural and functional features of cheese (Plessas et al., 2012; Settanni & Moschetti, 2010). Currently, probiotics such as *Lactobacillus*, *Bifidobacterium*, *Pediococcus*, *Staphylococcus*, and *Enterococcus* are added to cheese cultures to endow products beneficial to human health while inhibiting the growth of detrimental microorganisms that compromise food safety (Eugster et al., 2019; Heo et al., 2020; Ortakci et al., 2015; Peralta et al., 2016; Speranza et al., 2018). Such initiatives have altered our perception of cheese as follows: cheese is increasingly being regarded as a probiotics containing biomatrix that supports long‐term viability of probiotic organisms for maximal health benefits.

*Pediococcus acidilactici* is a Gram‐positive facultative anaerobic lactic acid bacterium. In recent studies, various *P. acidilactici* strains with potential probiotic value have been identified (Abbasiliasi et al., 2017; Akmal et al., 2019; Gupta & Sharma, 2017). *Pediococcus acidilactici* AS185 was isolated from traditional Chinese farmers' soybean paste and deposited in the China Center for Type Culture Collection (CCTCC) as M20181124 (Wang et al., 2019). This novel strain displayed significant functional characteristics, including efficient prevention and alleviation of early atherosclerosis in rats by regulating the levels of serum lipids, inflammatory adhesion molecules, and inflammatory factors (Wang et al., 2019). Yet no trials have been performed on this strain in humans; thus, this requires further clinical investigation. This study aimed to evaluate the performance of *P. acidilactici* AS185 as an adjunct culture for the production of probiotic cheddar cheese and assess the effects of this probiotic on the quality, physicochemical characteristics, and flavor development of cheese and viability of the microorganisms. Also, the effect of the cheese matrix on the viability of *P. acidilactici* AS185 during simulation of the passage through gastrointestinal tract was assessed.

## 2 MATERIALS AND METHODS

### 2.1 *Pediococcus acidilactici* AS185 biomass production

An adjunct culture of a potential probiotic *P. acidilactici* AS185 cell suspension was prepared as follows: pure culture of *P. acidilactici* AS185 was activated by inoculation in M17 medium (Coolaber, Beijing, China) at 37°C for 16 h. Then, the cell pellet was harvested by centrifugation of the culture at 5000*g* at 4°C for 10 min, washed with phosphate‐buffered saline (PBS, pH 7.2), and resuspended in PBS. The count of cells was adjusted (~10 log CFU/ml) by measurement of optical density at a wavelength of 600 nm (OD600) using a microplate reader (SpectraMax ABS Plus, Molecular Devices, America). And the cell suspension was stored at 4°C for subsequent use.

### 2.2 Manufacture of cheddar cheese

Cheddar cheese was manufactured, as described previously (Ong et al., 2007), with modifications, and all experiments were carried out in triplicate. For making each batch of cheese, 20 L of cow's milk was pasteurized at 63°C for 30 min and cooled to 37°C. Then, it was divided into two equal parts (for control and probiotic cheese varieties) and used for cheddar cheese manufacture. The following protocols were used to manufacture two types of cheddar cheese varieties: (i) a control cheese using 0.3 g commercial starter culture (R707; *Lactococcus lactis* subspecies *cremoris* and *Lactococcus lactis* subsp. *lactis*; Chr. Hansen, Denmark); (ii) a probiotic cheese using 0.3 g commercial starter culture and 100 ml of adjunct culture (*P. acidilactici* AS185 cell suspension). After 60 min postinoculation of starter cultures, CaCl_2_ (1.0 g) and rennet (CHY‐MAX Power NB, 0.14 g, Chr. Hansen, Denmark) were added to the pasteurized milk, and the mixture was allowed to stand at 37°C for 35–50 min to form curd that was cut into cubes (1 cm^3^) and stirred slowly. To separate the whey, the curd was heated slowly to 42°C (1°C increase every 6 min) until the pH declined to 5.8. Next, the whey was drained off, and curds were cheddared at 42°C and reversed stacking once every 15 min, 5–7 times; the final pH was reduced to 5.3. Subsequently, the curds were milled and salted (2%, w/w) before transfer to cheese molds. Next, the curds were pressed at 0.2 MPa pressure for 1.5 h, followed by pressing at 0.5 MPa pressure overnight. The next day, cheese samples were vacuum packed and stored at 8°C for 90 days for ripening.

### 2.3 Physicochemical composition analysis

The physicochemical properties of the ripened cheese samples (fat, protein, moisture, and pH) were assessed after ripening at 8°C for 90 days. Fat and total protein contents were determined using the Rӧse‐Gottlieb and Kjeldahl methods, respectively (Association of Official Analytical Chemists, 2007). The moisture content of cheese varieties was determined by drying the cheese samples at 102°C to obtain constant weight. The pH of the cheese samples was measured using a digital pH meter (Testo205, Testo Ltd., Titisee‐Neustadt, Germany).

### 2.4 Texture profile analysis

The texture profile analysis (TPA) of cheese samples was performed using a TA.XTplus Texture Analyzer (Stable Micro Systems Ltd., Godalming, UK) after ripening at 8°C for 90 days as described previously (Bourne, 2002). Briefly, cheese samples (1.5 cm^3^ cubes) were placed at room temperature for 2 h, followed by TPA using a P/5 probe at the following settings: 10% strain, probe pretest speed of 1.0 mm/s, probe test speed of 1.0 mm/s, probe posttest speed of 5.0 mm/s, compression deformation distance of 10.0 mm, and trigger force of 0.2 N.

### 2.5 Microbiological analysis

The total number of lactococci in each cheese sample was determined using a viable bacteria count process, as reported by Meira et al. (2015) with slight modifications. Cheese samples (25 g) were shredded, ground, and blended with 225 ml of peptone water (0.1 g/100 ml). Next, the mixture was homogenized with a hand‐held homogenizer (DLAB, Beijing, China). The homogenized sample was serially diluted (from 10^−1^ up to 10^−8^ dilution) with 0.85% NaCl solution, and the dilutions were spread onto M17 agar plates and then incubated at 37°C for 48 h. Finally, the colonies were counted, and the counts were expressed as the log of mean CFU/g of cheese. The microbiological analyses were performed at 1, 30, 60, and 90 days.

### 2.6 Free amino acids analysis

The free amino acids (FAAs) of cheese samples were assessed using high‐performance liquid chromatography (HPLC 1260, Agilent, CA, USA), as described by Manca et al. (2020), with modifications. An equivalent of 30 mg freeze‐dried powder of cheese sample at each ripening time point (1, 30, 60, and 90 days) was solubilized in 0.02 mol/L hydrochloric acid. The supernatant was collected after centrifugation (6000*g* at 4°C for 5 min). The injection volume was 10 μl of this diluted solution. HPLC was conducted using the following conditions: an Alltima C18 column (4.6 mm × 250 mm × 5 μm); mobile phase A of 0.1 mol/L in 97% anhydrous sodium acetate acetonitrile solution (pH = 6.5); B: 80% acetonitrile aqueous solution; injection volume 10 μl; column temperature: 40°C; detection wavelength 254 nm. The quantification was performed against α‐aminobutyric acid as an internal standard. The amino acid standards were purchased from Anpu Experimental Technology Co., Ltd. (Shanghai, China).

### 2.7 Short‐chain fatty acid analysis

Short‐chain fatty acids (SCFAs) of cheese samples were assessed using a gas chromatograph–mass spectrometer (GC–MS 7890B‐7000D, Agilent, CA, USA) as described by García‐Villalba et al. (2012) with modifications. The freeze‐dried powder of the cheese sample (30 mg) at each ripening time point (1, 30, 60, and 90 days) was blended with 2 ml of 25% phosphoric acid aqueous solution, followed by swirling homogenization, extraction with ethyl ether, centrifugation (4000*g* at 4°C for 20 min), and extraction with ethyl ether phase to obtain a 2‐ml reserve volume. The injection volume was 1 μl, which was conducted under the following conditions: HP‐InnoWAX column (25 m × 0.20 mm × 0.40 μm); heating procedure: 100°C for 5 min, followed by an increase to 150°C (at 5°C/min) and then an increase to 240°C (at 30°C/min), and the temperature (240°C) was held for 30 min; injection temperature 240°C; carrier gas flow rate of 1.0 ml/min; no shunt. Mass spectrometry analysis conditions were as follows: ion source temperature of 200°C; transmission line temperature of 250°C; EI source (electron impact ion source) bombardment voltage of 70 eV. The SCFAs were identified by comparison to chemical standards (acetic acid, propionic acid, isobutyric acid, butyric acid, isovaleric acid, and *N*‐valeric acid [TMstandard, Beijing, China]), while 2‐ethylbutyric acid was used as the internal reference.

### 2.8 Sensory evaluation

The sensory evaluation of cheese samples after ripening for 90 days was conducted according to the method of Toba et al. (1991). The samples were cut into cubes of about 7 cm^3^ volume/cube and placed on a plate of white glass. Then, the samples were tempered to the ambient temperature (20 ± 2°C) and randomly assigned to 12 trained panelists (6 females and 6 males, 25–35 years old) in random order. Water was provided between samples for mouth washing. The panelists evaluated the cheese varieties for flavor and taste, color and appearance, body and texture, and overall acceptance on a scale of 1–10 points, with 1 corresponding to worst and 10 corresponding to best quality.

### 2.9 In vitro gastrointestinal tolerance assay

The simulated gastrointestinal tract test was conducted on 90‐day‐ripened control and probiotic cheese varieties, as described by Madureira et al. (2011) using specific simulated gastrointestinal conditions (Table 1). Simulated saliva was generated by adding 100 U/ml α‐amylase (Sigma, USA) to 1 mmol/L CaCl_2_, and the pH was adjusted to 6.9 with 1 mol/L NaHCO_3_. Simulated gastric juice was generated by adding 25 mg/ml pepsin (Sigma) to 0.1 mol/L HCl. Duodenal fluid was generated by adding 2 g/L of pancreatic fluid to 12 g/L of bile salts (Sigma), and the pH was increased until 5.0 by 0.1 mol/L of NaHCO_3_. Finally, 0.1 mol/L of NaHCO_3_ was used to increase the pH to 6.5 in the ileum. All enzyme solutions were prepared fresh, filter sterilized through 0.22‐μm membrane filters (Millipore, Billerica, MA) prior to use and maintained in an ice bath during the period of simulation prior to their gradual addition to the test samples (when appropriate). For digestion of cheese samples, first, the oral digestion step was simulated using simulated saliva, which was added to cheese at the rate of 0.6 ml/min for 2 min. Next, to simulate the esophagus–stomach step, simulated gastric juice was added to the cheese–liquid mixture 0.05 ml, and the pH was decreased to 2 using 1 mol/L HCl, followed by incubation for 90 min. In the duodenal step, the duodenal fluid was added to the mixture at 0.2 ml/mL of the mixture. Finally, the pH was increased to 6.5 using 0.1 mol/L NaHCO_3_. During each digestion step, 1 ml of liquid was removed, and viable lactococci counts were determined by preparing serial decimal dilutions with peptone water (0.1 g/100 ml), which were subsequently plated on M17 agar medium, and incubated at 37°C for 48 h. The count of viable lactococci cells was expressed as log colony‐forming units per gram (log CFU/g).

**TABLE 1:** Processing conditions used in each step of simulated digestion

Compartment	Simulated digestive juice	Stirring (r/min)	Final pH	Time (min)
Mouth	Saliva	200	6.9	2
Esophagus–stomach	Pepsin	130	5.5	10
			4.6	10
			3.8	10
			2.8	20
			2.3	20
			2.0	20
Duodenum	Pancreatin + bile salts	45	5.0	10
Ileum	–	45	6.5	90

### 2.10 Statistical analysis

Each experiment was performed in triplicate. Statistical analysis was performed using SPSS v19.0 (SPSS Inc., Chicago, IL, USA). All data were subjected to Student's *t* test, and analysis of variance (ANOVA) with Tukey's test was used to determine statistically significant of differences between the control and probiotic cheese varieties; *p* ≤ .05 was considered statistically significant. The data were expressed as the mean value of three replicates. Redundancy analysis (RDA) was performed on data obtained for TPA parameters, viable lactococci count, FAAs, and SCFAs using CANOCO 4.5 software.

## 3 RESULTS AND DISCUSSION

### 3.1 Physicochemical composition analysis of probiotic cheese

After 90 days of storage, the physicochemical composition of each cheese sample was determined. The respective values for fat, protein, and water were 33.14%, 24.64%, and 34.28% for control cheese, and 33.24%, 24.82%, and 34.08% for probiotic cheese, with insignificant differences in the compositional parameters between samples (*p* > .05). Moreover, the pH of both the control and probiotic cheese varieties was 4.72 (*p* > .05). The addition of *P. acidilactici* AS185 organisms did not alter the composition of cheddar cheese, which was consistent with the previous results (Ong et al., 2007; Stefanovic et al., 2018). Notably, on the first day, the pH of the control and probiotic cheese was 5.33 and 5.45, respectively. The products became more acidic during 90 days of ripening. Finally, the control cheese showed a pH value that was lower than the normal range of 5.1–5.2, and the probiotic cheese had the same pH value as the control cheese. This is probably due to the metabolic activity associated with starter, nonstarter lactic acid bacteria (NSLAB), and probiotic bacteria, which convert lactose to lactic acid and other organic acids. Also, there may be complementary mechanisms between them (Angelopoulou et al., 2017; Antonia et al., 2019; de Oliveira et al., 2012). These findings indicated that the addition of an adjunct culture of *P. acidilactici* AS185 to the cheddar cheese should not alter the physicochemical composition of the cheese produced using standard cheddar cheese‐making techniques.

### 3.2 Texture profiles of the probiotic cheese

Texture profile analyses of cheese are presented in Table 2. The hardness values of probiotic cheese varieties were significantly lower than those of control cheese varieties (*p* < .05), which could be attributed to probiotic‐induced protein hydrolysis that loosens the micelle structure of casein, resulting in decreased hardness and soft cheese texture (Voigt et al., 2012). Nevertheless, the addition of probiotic *P. acidilactici* AS185 did not affect the chewiness, cohesiveness, or resilience significantly (*p* > .05).

**TABLE 2:** TPA parameters for 90‐day ripened cheddar cheese

Sample	Hardness (g)	Chewiness (−)	Cohesiveness (−)	Resilience (g·s)
Control cheese	700.81 ± 4.69^a^	642.17 ± 6.43^a^	0.89 ± 0.02^a^	0.41 ± 0.08^a^
Probiotic cheese	635.16 ± 8.87^b^	637.31 ± 4.56^a^	0.87 ± 0.02^a^	0.35 ± 0.04^a^

*Note*: Data are expressed as the mean and standard deviation (±SD) of three trials of which each trial consisted of triplicate assays. Values in columns with different small letters are significantly different (*p* < .05).

### 3.3 Survival of *P. acidilactici* AS185 in probiotic cheese during ripening

**FIGURE 1:**
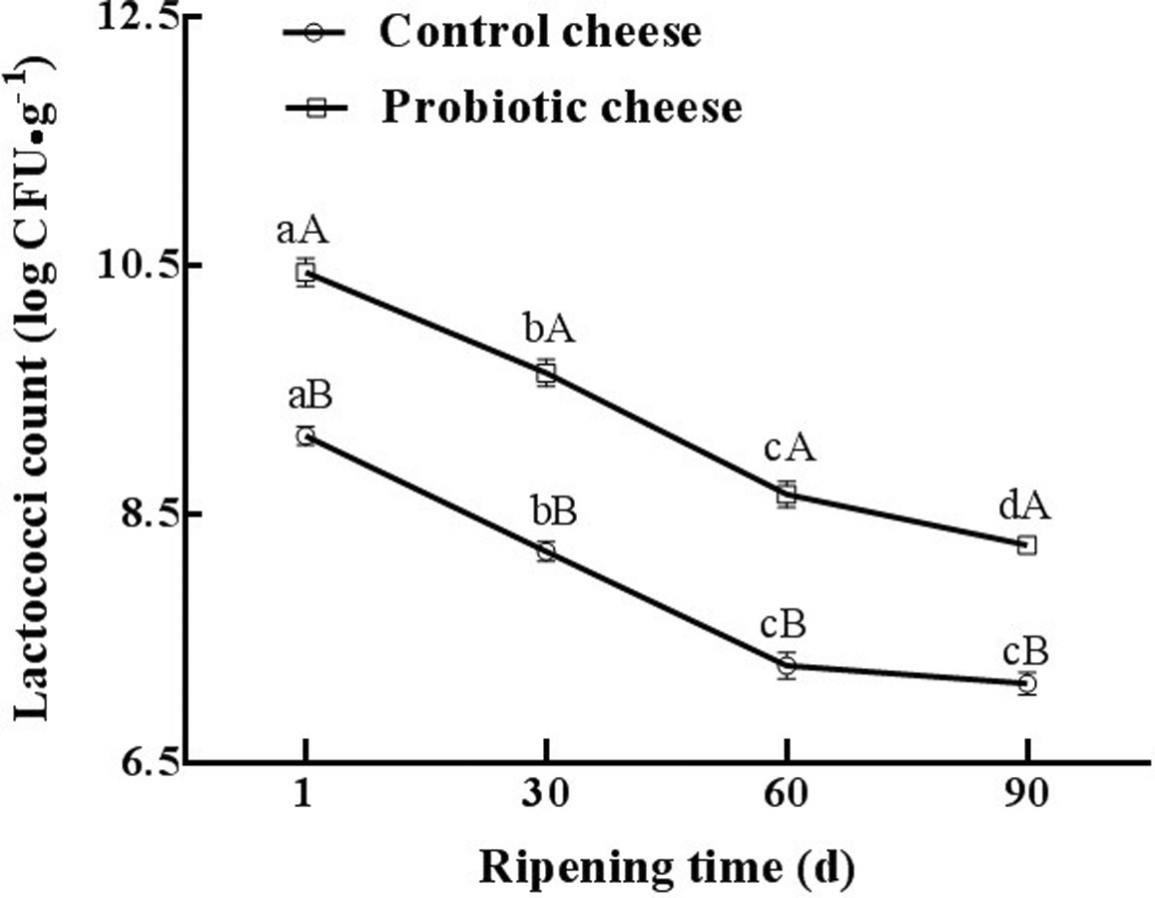
Changes in lactococci count during ripening of cheddar cheese varieties. Data are expressed as the mean and standard deviation (±SD) from three trials of which each trial consists of triplicate assays. (a–d) The same samples by different stages of ripening are significantly different (*p* < .05). (a, b) The same stages of ripening by different samples are significantly different (*p* < .05).

All cultures used to make cheddar cheese were lactococci (the commercial starter culture used in this study contained *L. lactis* subsp. *cremoris* and *L. lactis* subsp. *lactis*; the adjunct starter was *P. acidilactici* AS185 strain). The viable cell numbers of lactococci in cheese samples were counted during ripening (Figure 1). The results revealed that the counts of viable lactococci for each group decreased gradually with increasing duration of ripening because of the decrease in moisture level, increase in salt content, and the low ripening temperature. Yet, lactococci did not decrease below 10^7^ CFU/g. The final lactococci concentrations were 7.13 and 8.25 log CFU/g in control and probiotic cheese, respectively. Moreover, at each ripening time point (1, 30, 60, and 90 days), the number of viable lactococci in probiotic cheese was significantly higher than in control cheese (*p* < .05), which is likely because probiotic cheese contained the adjunct culture of *P. acidilactici* AS185 and the starter culture was added to both kinds of cheese. Thus, it was preliminarily concluded that *P. acidilactici* AS185 was viable in the probiotic cheese, and it could maintain a dynamic balance of cell autolysis and cell growth in the ripening environment of cheddar cheese. Unfortunately, due to the limitations of the laboratory conditions of this study, *P. acidilactici* AS185 population levels in cheddar cheese varieties were not estimated by PCR or other molecular techniques. At the same time, whether *P. acidilactici* AS185 as an adjunct starter influences the survival of the main starter by promoting symbiosis or antagonistic inhibition is also worthy of further study from multiple perspectives.

### 3.4 Free amino acids analysis of probiotic cheese during ripening

During cheddar cheese ripening, the probiotic adjunct starter and main starter strains constitute the microbial community within the cheese, wherein proteases produced by the probiotic organisms promote the production of FAAs and the development of cheese flavors (Antonsson et al., 2003). The metabolism of amino acids is strain specific (Peralta et al., 2016), while adjunct strains possess variable enzymatic activities that contribute to the overall proteolytic profile (Stefanovic et al., 2017). In this study, the 17 types of FAAs were detected (Table 3). As expected, the total amount of FAAs in the cheese varieties increased over‐ripening time, while different FAAs variably increased with ripening. Furthermore, the comparisons between nonprobiotic and probiotic cheese varieties at each ripening time point (1, 30, 60, and 90 days) revealed that probiotic contains significantly higher levels of FAAs, specifically Asp, Glu, His, Ala, Pro, Tyr, Val, Ile, and Lys compared to the control cheese (*p* < .05), with the peak of the overall amount of FAAs on day 90 (*p* < .05). It was assumed that because of the presence of *P. acidilactici* AS185 bacteria, they were mainly responsible for accelerating casein breakdown and contributing to the hydrolysis of medium‐sized peptides to amino acids. In this study, our results further confirmed the experimental conclusions of Ciocia et al. (2013) and Ewelina et al. (2018), who found that adding *Lactobacillus* probiotics could significantly influence the hydrolysis of secondary proteins to promote the production of FAAs as the cheese ripens. Moreover, it is worth noting that compared with the control cheese, the content of Leu and Phe in probiotic cheese was significantly increased after 90 days of ripening, which is also consistent with the results of Hannon et al. (2006). Leu and Phe are important indicators of cheddar ripeness. It was further indicated that the use of *P. acidilactici* AS185 as an adjunct culture could accelerate the ripening of cheese.

**TABLE 3:** Changes in contents of individual free amino acids during ripening of cheddar cheese varieties

FAA (mg/kg)	Control cheese				Probiotic cheese			
	1 day	30 days	60 days	90 days	1 day	30 days	60 days	90 days
Asp	39.17 ± 0.95^cB^	45.88 ± 0.68^bB^	63.67 ± 1.36^aB^	64.63 ± 0.79^aB^	42.16 ± 2.36^cA^	53.53 ± 3.20^bA^	86.31 ± 0.93^aA^	89.29 ± 0.98^aA^
Glu	188.90 ± 0.70^dB^	209.91 ± 0.74^cB^	214.47 ± 3.91^bB^	229.44 ± 0.88^aB^	300.56 ± 2.08^dA^	313.43 ± 1.47^cA^	511.83 ± 6.40^bA^	522.14 ± 2.20^aA^
Cys	57.51 ± 2.09^cA^	57.69 ± 2.32^cB^	92.25 ± 2.47^bB^	108.26 ± 2.73^aB^	39.25 ± 0.98^dB^	67.72 ± 1.56^cA^	97.12 ± 1.58^bA^	126.77 ± 1.57^aA^
Ser	80.52 ± 0.66^dA^	84.28 ± 0.96^cA^	88.62 ± 0.37^bA^	104.85 ± 0.52^aB^	19.90 ± 1.51^dB^	21.51 ± 0.97^cB^	47.55 ± 2.19^bB^	72.81 ± 2.51^aA^
Gly	9.28 ± 0.87^cB^	10.28 ± 0.11^cB^	32.89 ± 2.52^bA^	36.23 ± 1.71^aB^	12.15 ± 0.77^dA^	16.70 ± 0.26^cA^	25.25 ± 0.98^bA^	33.72 ± 1.65^aA^
His	39.97 ± 1.59^cB^	47.06 ± 1.53^bB^	51.25 ± 1.05^aB^	52.14 ± 1.67^aB^	55.11 ± 2.38^dA^	65.47 ± 2.44^cA^	71.19 ± 0.95^bA^	97.25 ± 1.78^aA^
Arg	125.17 ± 1.82^dA^	145.51 ± 4.61^cA^	246.45 ± 2.78^bB^	300.35 ± 0.73^aB^	93.89 ± 1.68^dB^	150.76 ± 5.10^cA^	317.56 ± 2.40^bA^	366.94 ± 2.24^aA^
Thr	8.02 ± 0.07^dA^	9.41 ± 0.39^cA^	12.83 ± 0.24^bB^	18.18 ± 0.15^aB^	7.50 ± 0.41^bA^	8.13 ± 0.13^bB^	24.10 ± 1.71^aA^	26.33 ± 2.39^aA^
Ala	65.31 ± 1.03^cB^	66.52 ± 0.39^cB^	71.46 ± 0.92^bB^	91.40 ± 0.79^aB^	74.98 ± 0.64^dA^	124.02 ± 1.40^bcA^	142.68 ± 57.74^bA^	205.01 ± 2.96^aA^
Pro	435.61 ± 4.91^dA^	448.29 ± 11.46^cB^	586.73 ± 2.93^aB^	553.51 ± 4.17^bB^	443.79 ± 3.31^dA^	522.93 ± 4.17^cA^	627.96 ± 3.53^bA^	661.16 ± 18.29^aA^
Tyr	119.05 ± 1.41^cB^	120.47 ± 4.53^cB^	166.46 ± 3.43^bB^	185.31 ± 2.76^aB^	125.33 ± 0.80^dA^	143.59 ± 1.43^cA^	182.99 ± 1.98^bA^	228.80 ± 1.07^aA^
Val	55.46 ± 1.00^dB^	61.14 ± 0.84^cB^	86.30 ± 1.02^bB^	101.46 ± 1.07^aB^	67.12 ± 4.57^dA^	102.27 ± 2.82^cA^	164.70 ± 3.29^bA^	206.30 ± 2.54^aA^
Met	12.79 ± 0.99^gA^	16.63 ± 0.46^fB^	25.91 ± 0.46^dB^	38.89 ± 0.28^aB^	12.51 ± 1.06^dA^	23.58 ± 1.12^cA^	62.90 ± 2.06^bA^	66.72 ± 0.54^aA^
Ile	20.29 ± 0.98^bB^	24.51 ± 0.89^bB^	46.28 ± 1.82^aB^	47.43 ± 4.27^aB^	24.66 ± 1.78^dA^	35.93 ± 1.53^cA^	66.51 ± 2.00^bA^	76.89 ± 1.31^aA^
Leu	300.27 ± 0.90^dA^	321.44 ± 0.91^cB^	534.20 ± 4.40^bB^	645.24 ± 2.93^aB^	230.17 ± 2.79^dB^	363.50 ± 4.92^cA^	681.98 ± 1.82^bA^	843.94 ± 3.98^aA^
Phe	242.39 ± 6.39^eA^	255.32 ± 1.00^dB^	406.93 ± 1.38^bA^	464.56 ± 3.99^aA^	153.40 ± 2.73^dB^	246.37 ± 4.39^cB^	377.08 ± 1.67^bB^	471.15 ± 16.13^aA^
Lys	99.03 ± 0.49^dB^	103.45 ± 1.98^cB^	116.69 ± 1.44^bB^	145.47 ± 2.96^aB^	155.81 ± 1.40^dA^	172.38 ± 11.59^cA^	206.54 ± 1.56^bA^	250.00 ± 3.54^aA^
Total	1898.64 ± 20.12^dA^	2027.77 ± 25.37^cB^	2843.38 ± 24.02^bB^	3187.32 ± 20.76^aB^	1858.74 ± 9.35^dB^	2431.83 ± 32.96^cA^	3702.71 ± 52.28^bA^	4336.74 ± 10.40^aA^

*Note*: Data are expressed as the mean and standard deviation (±SD) from three trials of which each trial consisted of triplicate assays. Values in rows with different small letters for same cheese during different ripening period are significantly different (*p* < .05). Values in rows with different capital letters for same ripening period between different cheese varieties are significantly different (*p* < .05).

### 3.5 Short‐chain fatty acids analysis of probiotic cheese during ripening

SCFAs play a major role in the development of cheese flavor. SCFAs produced during cheddar cheese ripening are directly responsible for aromatic flavors with low sensory thresholds while also acting as precursors for the production of other key flavor compounds, such as methyl ketones, alcohols, lactones, aldehydes, and esters (Leclercq‐Perlat et al., 2004). Herein, the main SCFAs detected in the control and probiotic cheese varieties are shown in Table 4. The SCFAs content of both types of cheese varieties changed significantly over 90 days of ripening (*p* < .05). The acetic acid content of the control and probiotic cheese varieties was significantly higher than that of the other SCFAs. During the 60‐day ripening period, the acetic acid content of probiotic cheese was significantly higher than that of the control cheese (*p* < .05); however, acetic acid content was reduced. There was no significant difference in the acetic acid content at 90 days of ripening (*p* > .05), which may be the reason why the cheese varieties had the same pH (as previously described in Section 3.1). The early acetic acid level peak could be attributed to strong early probiotic *P. acidilactici* AS185 activity that promoted lactic acid metabolism, leading to acetic acid production, while decreased acetic acid content during the late‐ripening stage may be due to the conversion of acetic acid to other flavor substances (Chen et al., 2012). These findings are consistent with the results obtained by Ong et al. (2007) and Eugster et al. (2019), wherein the probiotics increased the acetic acid levels in cheese varieties. In the late stage of ripening (60–90 days), the acetic acid content of both types of cheese varieties increased (20–40 mg/kg), isobutyric acid and butyric acid contents were also high (4–5.5 mg/kg), and their levels in probiotic cheese were significantly higher than those in the control cheese (*p* < .05). Butyric acid is one of the sources of the rich, creamy, and unpleasant smell of cheese. Butyric acid can give cheese juiciness and creaminess, but the threshold of butyric acid is low, and it has an unpleasant flavor at high concentrations, which will adversely affect the flavor quality of cheese. Atasoy and Tuerkoglu (2009) demonstrated that cheddar cheese with the best flavor contained 4.5–5.0 mg/kg butyric acid and 2.0–2.5 mg/kg caproic acid, as found here. Taken together, these results indicated that probiotic *P. acidilactici* AS185 participates in cheese flavor development and promotes the role of acetic acid and butyric acid in cheese flavor development, while the other SCFAs were low as reported by Moreira et al. (2018).

**TABLE 4:** Changes in contents of short‐chain fatty acids during ripening of cheddar cheese varieties

SCFAs (mg/kg)	Control cheese				Probiotic cheese			
	1 day	30 days	60 days	90 days	1 day	30 days	60 days	90 days
Acetic acid	22.80 ± .0.29^bB^	22.83 ± 0.31^bB^	24.51 ± 0.14^aB^	23.05 ± 0.03^bA^	32.70 ± 0.18^cA^	34.23 ± 0.37^bA^	39.73 ± 0.28^aA^	23.49 ± 0.42^dA^
Propionic acid	1.26 ± 0.13^dA^	2.14 ± 0.08^cA^	2.97 ± 0.07^bA^	3.60 ± 0.07^aA^	1.39 ± 0.04^dA^	2.15 ± 0.05^cA^	2.91 ± 0.03^bA^	3.19 ± 0.03^aB^
Isobutyric acid	1.66 ± 0.14^dA^	2.03 ± 0.08^cA^	3.24 ± 0.06^bA^	5.10 ± 0.10^aB^	0.98 ± 0.08^dB^	1.43 ± 0.10^gB^	2.44 ± 0.13^dB^	5.51 ± 0.04^aA^
Butyric acid	1.55 ± 0.05^dB^	3.08 ± 0.09^cA^	3.68 ± 0.04^bB^	4.21 ± 0.02^aB^	1.76 ± 0.01^gA^	2.85 ± 0.02^cB^	4.06 ± 0.01^bA^	5.22 ± 0.05^aA^
Isovaleric acid	0.53 ± 0.07^cA^	0.57 ± 0.06^cA^	0.83 ± 0.08^bB^	0.99 ± 0.06^aB^	0.52 ± 0.04^dA^	0.61 ± 0.04^cA^	1.17 ± 0.05^bA^	1.47 ± 0.06^aA^
Valeric acid	0.27 ± 0.04^cA^	0.28 ± 0.04^cB^	0.41 ± 0.05^bB^	0.73 ± 0.04^aB^	0.32 ± 0.07^cA^	0.39 ± 0.03^cA^	0.69 ± 0.07^bA^	1.52 ± 0.07^aA^

*Note*: Data are expressed as the mean and standard deviation (±SD) from three trials of which each trial consisted of triplicate assays. Values in row with different small letters for same cheese during different ripening period are significantly different (*p* < .05); Values in row with different capital letters for same ripening period between different cheese varieties are significantly different (*p* < .05).

### 3.6 Redundancy analysis of probiotic cheese

**FIGURE 2:**
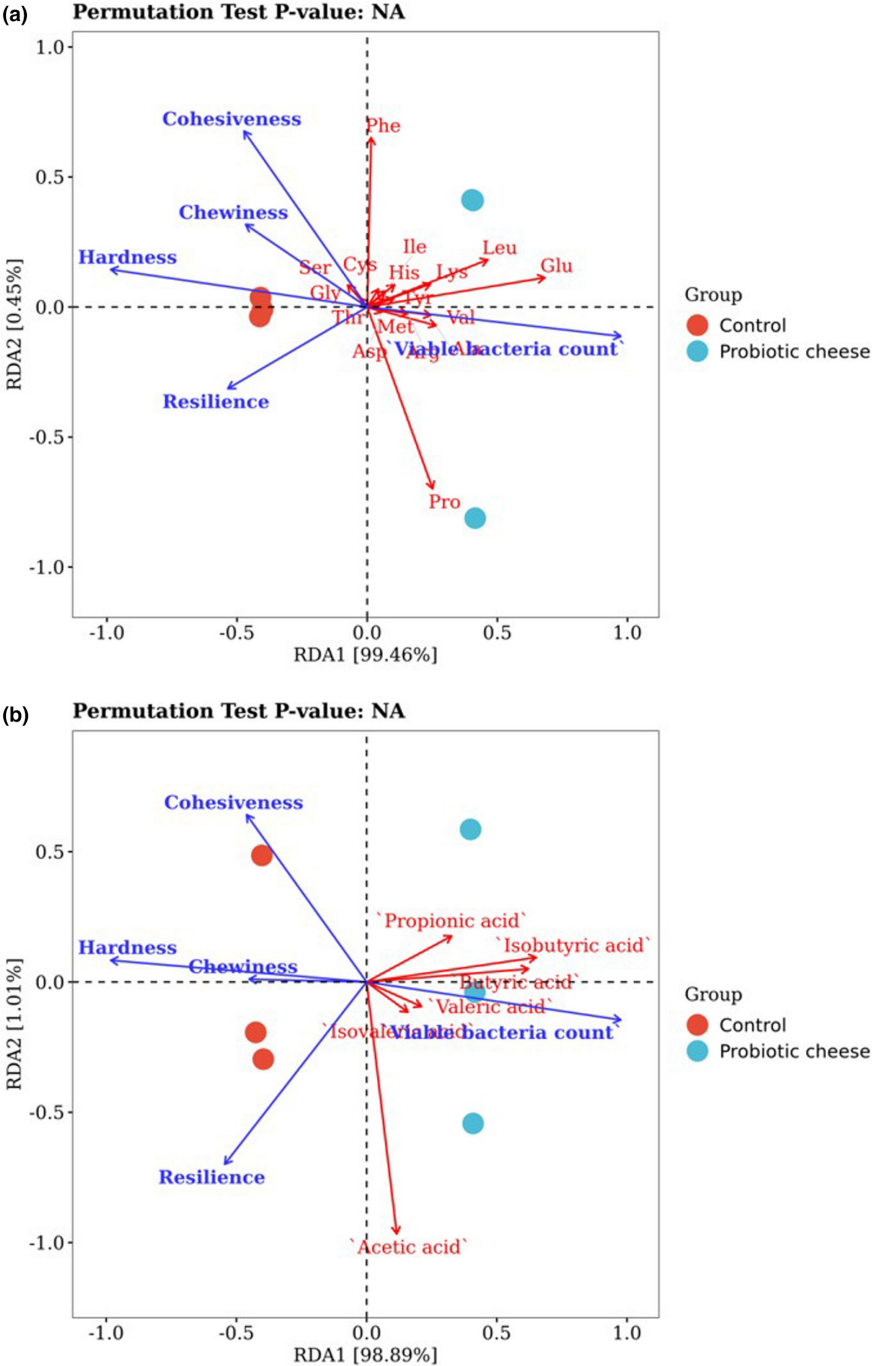
RDA analysis of TPA parameters, lactococci count, FAAs, and SCFAs in control and probiotic cheese varieties at 90 days of ripening. (a) RDA analysis of TPA parameters, lactococci count, and FAAs. (b) RDA analysis of TPA parameters, lactococci count, and SCFAs.

RDA was used to investigate associations of TPA parameters (hardness, chewiness, cohesiveness, and resilience), lactococci count, FAAs, and SCFAs in control and probiotic cheese varieties on day 90 of ripening (Figure 2). As shown in Figure 2a, RDA1 and RDA2 contributed to 99.46% and 0.45% of the total variance, respectively. Notably, *P. acidilactici* AS185 was associated with FAAs and lactococci count but not with TPA parameters. A lactococci count was required for the formation of FAAs, such as Glu, Leu, Lys, Thr, Val, Arg, and Ala, in probiotic cheese varieties, thereby demonstrating that *P. acidilactici* AS185 probably stimulated the catabolism of proteins, which in turn increases the level of FAAs liberated from peptides. Figure 2b shows the associations of SCFAs with texture and lactococci count, wherein RDA1 and RDA2 comprised 98.89% and 1.01% of the total variance, respectively. The lactococci count was also related to the SCFAs, such as isobutyric, valeric, butyric, and isovaleric acids. Interestingly, the textural properties of cheese varieties were independent of SCFAs. Therefore, we deduced that cheese lactococci count is correlated with FAAs and SCFAs levels in the cheese varieties since *P. acidilactici* AS185 plays a key role in cheese flavor development.

### 3.7 Sensory analysis of probiotic cheese

The sensory evaluation results for cheese varieties on day 90 of ripening are shown in Table 5. The results revealed significant differences (*p* < .05) in flavor and texture between the control and probiotic cheese varieties. This may also be related to the production of deaminases, decarboxylases, peptidases, or transaminases by *P. acidilactici* AS185, which further promote the catabolism of proteins, producing short peptides and FAAs. These can act as precursors and be biochemically converted into specific cheese‐flavor substances. In this study, the probiotic cheese on day 90 of ripening had the highest Leu content (Table 3). Leu is one of the important precursors of flavor compounds that has a key role in the formation of cheese flavor. Leu can be biosynthesized by transaminases and degraded by Strecker to produce 3‐methylbutyral, which gives the cheese a nutty flavor (McSweeney & Sousa, 2000; Yvon & Rijnen, 2001). This may be the main reason for the high score of probiotic cheese flavor. Strikingly, probiotic cheese such as gouda cheese with adjunct cultures of *Bifidobacterium longum* and *B. lactis*, possessed superior flavor and more complex aroma than the control cheese (McBrearty et al., 2001). Some studies reported that the addition of probiotics altered the sensory properties of the cheese slightly (Chen et al., 2019; Saiki et al., 2018), though no differences were detected in the appearance and overall sensory properties between the two types of cheese varieties (Table 5). Nevertheless, compared to control cheese, probiotic cheese were preferred by the panelists, as reflected by high‐quality scores. Taken together, these results suggested that the addition of probiotic does not affect the overall sensory properties of cheese rather improves the flavor.

**TABLE 5:** Sensory scores for 90‐day ripened cheddar cheese varieties

Sample	Sensory parameters			
	Flavor and taste	Color and appearance	Body and texture	Overall acceptance
Control cheese	7.93 ± 0.76^b^	9.10 ± 0.36^a^	7.88 ± 0.43^b^	8.56 ± 0.52^a^
Probiotic cheese	9.37 ± 0.30^a^	9.20 ± .035^a^	8.73 ± 0.33^a^	8.96 ± 0.50^a^

*Note*: Data are expressed as the mean and standard deviation (±SD) from three trials of which each trial consists of triplicate assays. Values in columns with different small letters are significantly different (*p* < .05).

### 3.8 Analysis of in vitro gastrointestinal tolerance of probiotic cheese

**FIGURE 3:**
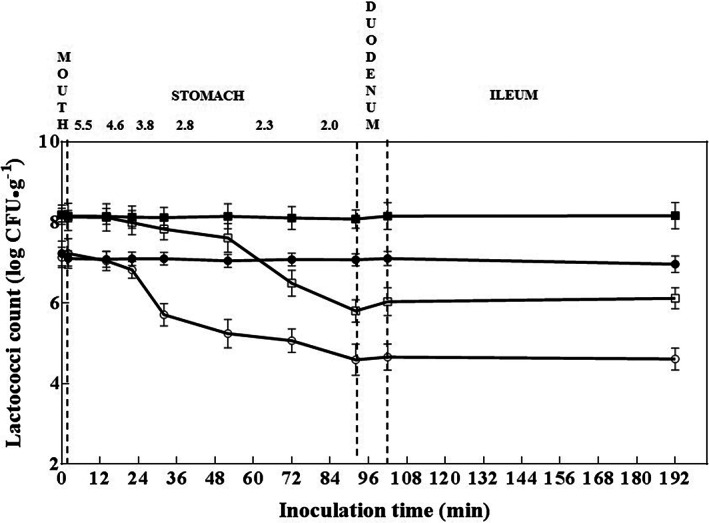
Lactococci count of control (○, ●) and probiotic cheese (□, ■) at 90 days of ripening, for cheese varieties exposed (○, □) or not exposed (●, ■) to simulated gastrointestinal conditions throughout incubation time; pH values are indicated in the upper left corner.

Cheese is a highly effective carrier of probiotic bacteria within the gastrointestinal tract (Manning & Gibson, 2004) due to its buffering and shielding functions that protect the organisms within from exposure to harsh conditions in the stomach and small intestine (Grajek et al., 2005). Herein, lactococci in cheese during various stages of simulated gastrointestinal digestion were counted (Figure 3). No decrease in viable counts was detected in either type of cheese (probiotic or nonprobiotic) after exposure to initial digestive conditions in the mouth, which were simulated using saliva solution prepared with α‐amylase, a dominant active compound in natural saliva (Humphrey & Williamson, 2001). Thus, our results indicated that the viability of cheese probiotic lactococci was not affected by α‐amylase and pH conditions in the oral cavity. Conversely, the exposure of cheese varieties to conditions prevailing in the esophagus and stomach decreased the lactococci number in both types of cheese varieties when the pH dropped to 2.8 in the presence of pepsin. Specifically, after 92 min of digestion but prior to exposure to simulated ileum conditions, viable cell numbers in control and probiotic cheese varieties were approximately 2.5 log cycles and 2.0 log cycles lower than those during the oral digestion stage, respectively. However, the pH during the simulated ileum stage was 6.5, which was close to neutral and had little effect on the viable cell counts for both cheese varieties; thus, the viable cell count of the probiotic cheese remained consistently higher than that of the control cheese. Importantly, our results revealed that in the simulated stomach and duodenum, the number of lactococci in the cheese varieties decreased, while the conditions in the simulated oral cavity had little effect on the survival of lactococci within the cheese varieties. This means that this culture strains and *P. acidilactici* AS185 strain were not affected by mouth conditions; they can reach the lower gastrointestinal environment. Yet, during exposure to conditions prevailing in the stomach, they suffered a decrease in viable numbers when pH reached 2.0, and this number increased when the organisms entered the simulated duodenal conditions. Moreover, the number of viable bacteria in probiotic cheese increased significantly. The increase in pH appeared to be favorable for the survival of these strains. This subsequent rapid increase in the cell count was certainly not an outcome of cell division but may be a recovery of sublethally injured cells – since previous steps had led to viable number reduction. This phenomenon is supported by a study wherein some lactic acid bacteria enter a state of false death or dormancy during exposure to gastric acid, and the cells can recover after a pH rise (Ruiz‐Moyano et al., 2008; Verruck et al., 2017). Another study showed that the number of viable bacteria declined during the simulated duodenal step and never recovered due to more severe damage inflicted by bile salts compared to the other treatments (Maragkoudakis et al., 2006). On the other hand, probiotic cheese appears to be the most resistant to transit throughout the gastrointestinal tract, as the number of viable bacteria in the probiotic cheese was consistently significantly higher than that in the control cheese. Herein, for *P. acidilactici* AS185, the protection effect of cheese was also clearly shown throughout all steps of simulated digestion via comparison with their performance in control cheese. But the specific survival status of *P. acidilactici* AS185 strain in cheese still needs to be analyzed by other molecular‐assisted techniques.

## 4 CONCLUSION

The current data suggested that adding *P. acidilactici* AS185 during cheese production must not adversely affect the physicochemical properties and texture of cheddar cheese. Moreover, strain *P. acidilactici* AS185 was viable in the cheese ripening stages, promoting the catabolism of proteins and fats, which in turn produced more FAAs and SCFAs, and improved the flavor and taste of cheese. In addition, the cheese matrix appears to favorably influence the survival of strain *P. acidilactici* AS185 during digestion. To sum up, *P. acidilactici* AS185 adjunct culture in cheddar cheese production supports the formation of a high‐quality product with characteristic flavor and texture. Therefore, *P. acidilactici* AS185 is a promising strain for producing probiotic cheese products.

## FUNDING INFORMATION

Jilin Academy of Agricultural Sciences, Grant/Award Number: KYJF2021JQ008. China Agriculture Research System of MOF and MARA, Grant/Award Number: CARS36.

## CONFLICT OF INTEREST

The authors declare that there are no conflicts of interest regarding the publication of this article.

## Data Availability

The data that support the findings of this study are available from the corresponding author upon reasonable request.
